# In Vitro Evaluation of Smart and pH-Sensitive Chondroitin Sulfate/Sodium Polystyrene Sulfonate Hydrogels for Controlled Drug Delivery

**DOI:** 10.3390/gels8070406

**Published:** 2022-06-25

**Authors:** Muhammad Suhail, I-Hui Chiu, Ming-Chia Hung, Quoc Lam Vu, I-Ling Lin, Pao-Chu Wu

**Affiliations:** 1 School of Pharmacy, Kaohsiung Medical University, 100 Shih-Chuan 1st Road, Kaohsiung 80708, Taiwan; u108830004@kmu.edu.tw (M.S.); u110530002@kmu.edu.tw (I.-H.C.); u110530001@kmu.edu.tw (M.-C.H.); 2 Department of Clinical Pharmacy, Thai Nguyen University of Medicine and Pharmacy, 284 Luong Ngoc Quyen Str., Thai Nguyen City 24000, Vietnam; vuquoclam@tump.edu.vn; 3 Department of Medicine Laboratory Science and Biotechnology, College of Health Science, Kaohsiung Medical University, Kaohsiung 80708, Taiwan; linili@kmu.edu.tw; 4 Department of Medical Research, Kaohsiung Medical University Hospital, Kaohsiung 80708, Taiwan; 5 Drug Development and Value Creation Research Center, Kaohsiung Medical University, Kaohsiung 80708, Taiwan

**Keywords:** pH-sensitive hydrogels, ibuprofen, swelling, dissolution

## Abstract

Ibuprofen is an antipyretic and analgesic drug used for the management of different inflammatory diseases, such as rheumatoid arthritis and osteoarthritis. Due to a short half-life and rapid elimination, multiple doses of ibuprofen are required in a day to maintain pharmacological action for a long duration of time. Due to multiple intakes of ibuprofen, certain severe adverse effects, such as gastric irritation, bleeding, ulcers, and abdominal pain are produced. Therefore, a system is needed which not only prolongs the release of ibuprofen but also overcomes the drug’s adverse effects. Hence, the authors have synthesized chondroitin sulfate/sodium polystyrene sulfonate–co-poly(acrylic acid) hydrogels by the free radical polymerization technique for the controlled release of ibuprofen. Sol-gel, porosity, swelling, and drug release studies were performed on the fabricated hydrogel. The pH-responsive behavior of the fabricated hydrogel was determined by both swelling and drug release studies in three different pH values, i.e., pH 1.2, 4.6, and 7.4. Maximum swelling and drug release were observed at pH 7.4, as compared to pH 4.6 and 1.2. Similarly, the structural arrangement and crosslinking of the hydrogel contents were confirmed by Fourier transform infrared spectroscopy (FTIR). Scanning electron microscopy (SEM) evaluated the hard and irregular surface with a few macrospores of the developed hydrogel, which may be correlated with the strong crosslinking of polymers with monomer content. Similarly, thermogravimetric analysis (TGA) and differential scanning calorimetry (DSC) demonstrated the high thermal stability of the formulated hydrogel, as compared to pure polymers. A decrease in the crystallinity of chondroitin sulfate and sodium polystyrene sulfonate after crosslinking was revealed by powder X-ray diffraction (PXRD). Thus, considering the results, we can demonstrate that a developed polymeric network of hydrogel could be used as a safe, stable, and efficient carrier for the controlled release of ibuprofen.

## 1. Introduction

Hydrogel is a three-dimensional polymeric structure which absorbs a greater amount of water while maintaining its dimensional stability [1,2]. Hydrogels are hydrophilic in nature, thus have the ability to preserve a high volume of water. Due to unique characteristics including greater stability, hydrophilicity, biocompatibility, biodegradability, and low toxicity, hydrogels are used for different purposes, i.e., targeted, topical, localized drug delivery systems [3,4], cellular immobilization, tissue engineering [5], wound dressing [6], and diagnosis. The response of hydrogel to external stimuli, i.e., pH, magnetic fields, temperature, electric fields, biological molecules, and ionic strength of solution, is another astounding characteristic of hydrogel [7] which further enhances its use, especially in controlled drug delivery systems [8]. Various natural and synthetic polymers are employed in combination with various monomer(s) for the synthesis of polymeric networks of hydrogel while using a number of crosslinking procedures [9,10]. A tissue-like structure is adopted by hydrogel in its swelled form, which demonstrates the soft and rubbery nature of hydrogel. Furthermore, hydrogel protects the loaded drug from the unfavorable environment of the body [11,12,13].

Chondroitin sulfate (Cs) is composed of D-glucuronic acid connected to N-acetyl-D-galactosamine. It is a natural polysaccharide polymer. Its solubility in water is high. The biological source of Cs is the cartilage of animals [14]. Cs protects the connective tissues by regulating the water content, thus, it has been used in the treatment of severe disorders such as atherosclerosis, osteoarthritis, and thrombosis for a long time [15,16]. Cs absorbs a greater quantity of water because it contains COO− and –SO_3_− functional groups, thus, it is used widely in hydrogel preparation [17]. Polystyrene sulfonate is a polydisperse linear sulfonated, antimicrobial, and non-cytotoxic polymer. The solubility of polystyrene sulfonate is very high in water and aqueous alkaline solutions, if its molecular weight is 500–800 kDa. Sodium polystyrene sulfonate (Sps) is the sodium salt of polystyrene sulfonate, which is used for various purposes, including cosmetics, multilayer membranes of polyelectrolyte for controlled drug release, exchange membranes of proton for fuel cells, and medicines to manage hyperkalemia (abnormally high potassium or lithium levels in the blood). Because of SO_3_, Sps absorbs a greater amount of water, and thus is frequently used in the development of hydrogels [18,19]. Acrylic acid (Aca) is a synthetic pH-sensitive monomer, which shows maximum swelling at high pH values as compared to low pH values, and thus is used mostly in the fabrication of pH-sensitive hydrogels. Because of COOH functional groups, Aca increases the sensitivity of the medium, thus showing its protonation and deprotonation in low- and high-pH media. Due to its pH-sensitive nature, maximum swelling and drug release are detected at neutral pH, while low in an acidic medium, thus protecting the encapsulated drug from the acidity of the stomach. Therefore, Aca is used in both pharmaceutical and biomedical fields [20].

Ibuprofen (Ibu) is a 2-(4-Isobutylphenyl) propionic acid, which is used as an antipyretic and analgesic for the management of various inflammatory diseases [21]. It may be employed for short duration therapy, such as for headaches, or chronic therapy, such as for rheumatoid arthritis and osteoarthritis. It has a very low solubility of nearly 1 mg/mL [22], whereas 100% absorption occurs approximately with a high plasma concentration around 60–120 min after oral intake [23,24]. Ibu is excreted from systemic circulation very rapidly because of its short half-life (2 h). Therefore, multiple doses of Ibu are needed to achieve effective pharmacological action for a prolonged time [25]. Furthermore, it is more important to maintain the analgesic effect in the management of chronic disorders, rather than the rapid onset of action. Considering the multiple doses of Ibu needed several times a day in order to maintain a constant therapeutic level, various adverse effects, including gastric irritation, gastric toxicity, bleeding, ulcers, and abdominal pain are generated [26]. Hence, in order to overcome these adverse effects and achieve effective pharmacological action of Ibu for extended periods of time, a prominent drug delivery system is needed, which can extend the release of Ibu in a controlled way. Therefore, different drug delivery systems were prepared for sustained/controlled delivery of Ibu. Sogias et al. (2012) developed chitosan-based tablets for sustained delivery of Ibu [27]. Alshehri and his coworkers developed natural macroporous sporopollenin exine capsules for controlled delivery of Ibu [28]. Similarly, Borovac et al. (2006) synthesized polyvinyl alcohol-based hydrogel beads for sustained release of Ibu [29]. However, a lot of work is still needed to overcome the adverse effects generated as a result of multiple intakes of Ibu. Due to its unique properties, hydrogel is regarded as one of the most suitable drug delivery systems for the controlled delivery of therapeutic agents [30,31]. Therefore, in recent investigations, the authors have tried to prepare chondroitin sulfate/sodium polystyrene sulfonate-based hydrogels for the controlled release of Ibu.

The novelty of the recently developed hydrogel was based on the usage of both natural and synthetic polymers with a pH-sensitive synthetic monomer. Aca was crosslinked with Cs and Sps, and thus increased the pH sensitivity of the developed hydrogel, which not only protects the drug from acidic environments, but also protects the stomach from the adverse effects produced as a result of multiple intakes of Ibu. Thus, the unique properties of Aca have especially enhanced its use in targeted and controlled drug delivery systems. The main advantage of the developed hydrogel is not only concerned with the controlled release of Ibu, but it can also be employed for the controlled delivery of different therapeutic agents. A series of studies was performed for the developed hydrogels. Sol-gel analysis revealed high gel fraction and low sol fraction with an increase in the concentration of hydrogel contents. The penetration capability of the aqueous medium through the hydrogel networks was determined by a porosity study. High swelling and drug release were detected at pH 7.4, as compared to pH 1.2 and 4.6. Similarly, due to maximum swelling, high loading of the drug was observed by the prepared hydrogels. Likewise, FTIR confirmed the crosslinking among hydrogel contents, whereas SEM indicated an irregular surface with a few large pores. These pores are responsible for the penetration of water through the hydrogel networks. Thermal analysis presented greater thermal stability for the fabricated hydrogel, as compared to unreacted polymers. Similarly, crystallinity of the Cs and Sps was reduced due to crosslinking among hydrogel contents, as indicated by PXRD analysis of the developed hydrogel. Conclusively, we can demonstrate that the prepared network of hydrogel is the most suitable and promising carrier for the controlled delivery of Ibu.

## 2. Results and Discussion

### 2.1. Fabrication of Polymeric Hydrogels

**Figure 1:**
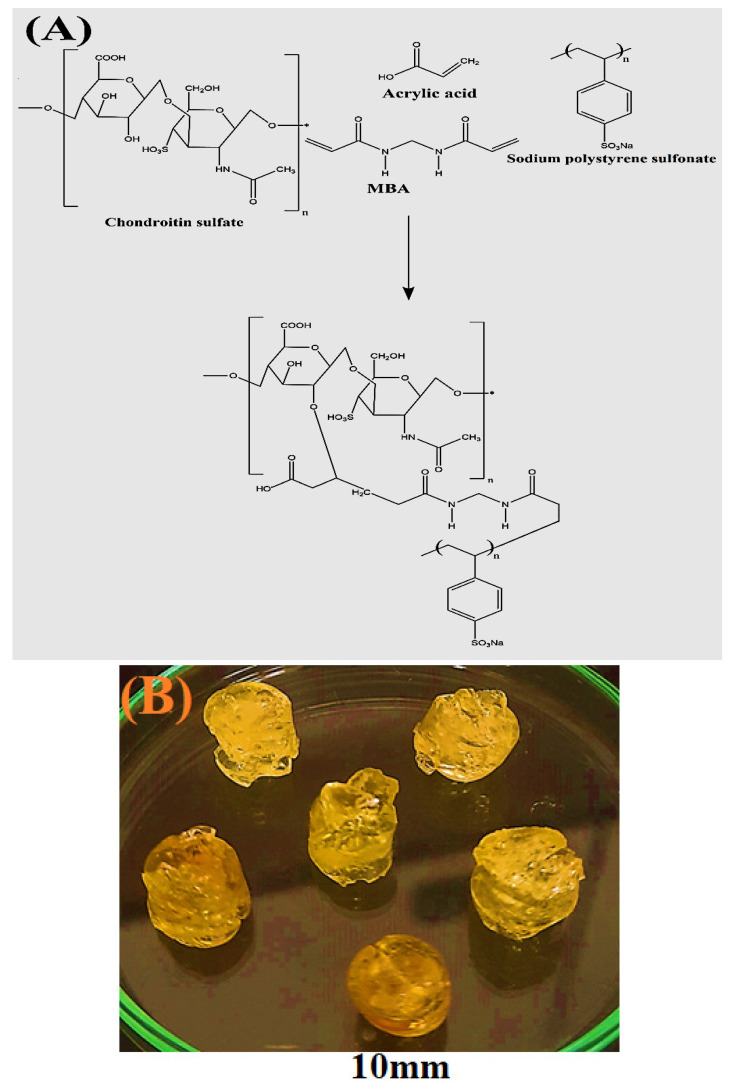
(**A**) Proposed chemical structure and (**B**) physical appearance of Cs/SpsCpAca hydrogels.

The free radical polymerization technique was employed for the development of hydrogel, which is used commonly for the preparation of different types of hydrogels. A series of nine formulations was formed by the crosslinking of various combinations of Cs, Sps, and Aca in the presence of Mba and Aps. A greater number of free radicals was produced by increasing the concentration of Cs, Sps, and Aca, which were initiated and crosslinked by Aps and Mba, respectively. Different studies, including porosity, sol-gel analysis, drug loading, polymer volume fraction, swelling, and dissolution, were performed for the developed hydrogels. Similarly, characterizations such as FTIR, SEM, DSC, TGA, and PXRD were carried out for the fabricated networks of hydrogel. The proposed chemical structure and physical appearance of the prepared hydrogel are shown in Figure 1A,B.

### 2.2. Sol-Gel Analysis

**Figure 2:**
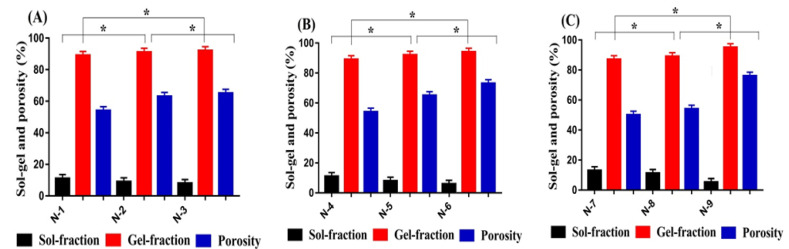
Effect of (**A**) Cs, (**B**) Sps, and (**C**) Aca on sol-gel and porosity of Cs/SpsCpAca hydrogel.

During the polymerization technique, a soluble part of hydrogel contents is not crosslinked, while an insoluble part is crosslinked. The soluble and uncrosslinked part of the hydrogel is recognized as sol-fraction, while gel fraction is the insoluble and crosslinked part of the hydrogel. Sol-gel analysis was performed for the developed hydrogel with the purpose of evaluating its sol and gel fraction. Both sol and gel fractions were influenced by the different compositions of Cs, Sps, and Aca, as shown in Figure 2A–C. A rise in gel fraction was observed with the increasing compositions of Cs and Sps (Figure 2A,B). The higher the amount of Cs and Sps, the greater the availability of free radicals, thus higher the gelation and polymerization of both polymers with monomer. Similarly, an increase in the composition of Aca (Figure 2C) led to the generation of a high number of free radicals, which were crosslinked with the polymers, and thus, a resulting increase in gelation was observed. The same effects of hydrogel contents were observed by Khanum and her coworkers [32], which further support our findings. In contrast to gel fraction, a drop was seen in sol fraction with the increasing compositions of Cs, Sps, and Aca, due to the inverse relation with gel fraction. Decrease in sol fraction leads to an increase in gel fraction, and vice versa [33]. *p* values were found less than 0.05 for all formulation, which were identified with (*) as shown in Figure 2A–C. 

### 2.3. Porosity Study

Both swelling and drug loading are dependent on the porosity of a hydrogel. Channels are provided by the porous surface of the hydrogel for the penetration of water into its polymeric networks. Thus, we can demonstrate that the higher the porosity of the hydrogel, the greater the swelling and drug loading. Hydrogel contents affected the porosity of the hydrogel by changing their composition from lower to upper values. Porosity was increased with enhancement in the compositions of Cs, Sps, and Aca, as shown in Figure 2A–C. The reason may be related to the high viscosity of the reaction mixture. Escalation in the viscosity of the reaction mixture occurred as the composition of Cs, Sps, and Aca was enhanced during the process of free radical polymerization. The high viscosity led to the prevention of bubbles’ evaporation, and as a result, interconnected channels were formed which enhanced the water penetration into the hydrogel networks. Hence, an increase in porosity was observed, and vice versa [34].

### 2.4. Polymer Volume Fraction

Polymer volume fraction was estimated for the fabricated hydrogel in buffer solutions of pH 1.2, 4.6, and 7.4, respectively, as indicated in Table 1. Polymer volume fraction was achieved higher at pH 1.2, while at pH 4.6 and 7.4, low polymer volume fraction was obtained for all formulations of the developed hydrogel. A change was seen in the fraction of polymer volume with the varying compositions of hydrogel contents. A reduction in the volume of the polymer was perceived with high compositions of polymers and monomer, i.e., Cs, Sps, and Aca. Thus, a decrease in the volume of the polymer at pH 7.4 and 4.6 was due to the higher swelling index of the fabricated hydrogel. The low fraction of polymer volume fraction at pH 4.6 and 7.4, while higher at pH 1.2, confirmed the maximum swelling of the fabricated hydrogel at high pH values while almost low at a low pH value [35].

### 2.5. Dynamic Swelling/Drug Loading and Drug Release Studies

**Figure 3:**
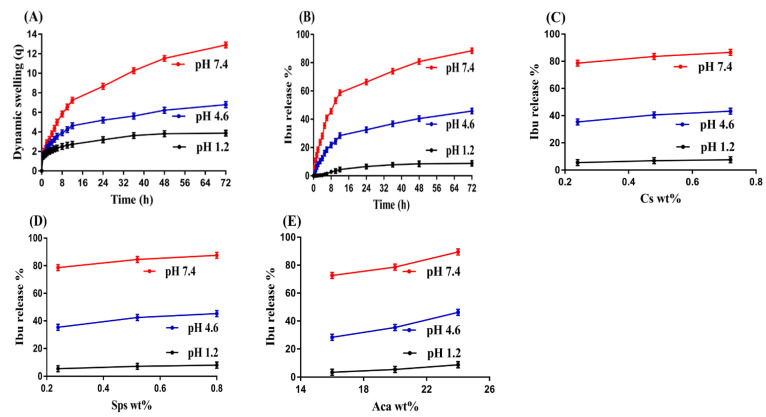
Effect of pH on (**A**) dynamic swelling and (**B**) percent of drug released from Cs/SpsCpAca hydrogel. Effect of (**C**) Cs, (**D**) Sps, and (**E**) Aca on percent of drug released from Cs/SpsCpAca hydrogel.

The pH sensitivity of the developed hydrogel was demonstrated by conducting swelling and drug release studies at three altered pH values, i.e., pH 1.2, 4.6, and 7.4, respectively. Maximum swelling and drug release were observed at pH 7.4 as compared to pH 4.6, while very low swelling and drug release were seen at pH 1.2, as indicated in Figure 3A,B. Cs contained SO_3_ and COOH functional groups, whereas Sps consisted of SO_3_, respectively. Similarly, Aca has COOH functional groups. Due to the deprotonation of functional groups of Cs, Sps, and Aca, greater swelling and release of the drug were detected at high pH values, especially at pH 7.4, as compared to pH 4.6. The same functional groups of polymers and monomers enhanced their charge density, and thus, strong electrostatic repulsive forces were produced. These forces caused in repulsion of the same charged groups and thus an expansion in the volume of hydrogel was detected, which further led to high swelling and release of the drug, and vice versa. Contrary to pH 4.6 and 7.4, very low swelling and drug release were detected at acidic pH 1.2. The possible reason for this is the protonation of functional groups of Cs, Sps, and Aca at low pH. The COOH and SO_3_ groups of Cs, Sps, and Aca formed conjugates with the counter ions by strong hydrogen bonding. Due to strong intermolecular forces, charge density of the same charged groups was reduced and thus a decrease in swelling and drug release was observed at pH 1.2. Hence, we can conclude from the discussion that swelling and drug release occurred in a pattern, i.e., pH 7.4 > 4.6 > 1.2, demonstrating the pH-sensitive nature of the developed hydrogel [36,37,38]. Different researchers have prepared a number of drug carrier systems for the extended release of ibuprofen. Martinez et al. prepared ibuprofen-loaded hydrogel and reported swelling and release of ibuprofen from hydrogel for 24 and 10 h, respectively [39]. Similarly, Sun and his coworkers developed hydroxyethyl cellulose-based hydrogels and reported swelling and sustained release of ibuprofen for 24 h [40]. In the current study, the authors prepared Cs/SpsCpAca hydrogels for the controlled delivery of Ibu. The swelling index and release of Ibu from the prepared polymeric network of hydrogel were reported for 72 h. Thus, comparing the results of the previous published studies with the current research work, we can see that the release of Ibu was prolonged for 72 h by the newly fabricated hydrogel with a high swelling index. Finally, we can conclude that the prepared hydrogel could be used as a promising carrier for controlled drug delivery.

Drug loading relies on swelling, which in turn depends on the porosity of the hydrogel. There is a direct relation between the drug loading and swelling index of hydrogels [41]. The higher the swelling index, the greater the loading of the drug, and vice versa. In our study, swelling, drug loading, and drug release were influenced by the increasing compositions of hydrogel contents, as shown in Table 1 and Table 2, and Figure 3C–E. An increase in Cs composition directly led to high swelling and drug loading of the hydrogel. The possible reason was attributed to the high charge density of functional groups of Cs, which produced high swelling, and thus an increase in loading and release of the drug was observed. Similarly, high compositions of Sps and Aca led to greater swelling, loading, and release of the drug due to an increase in their charge density, which caused in repulsion of the same charged groups. Thus, an enhancement in swelling, loading, and release of the drug was perceived [42,43,44,45,46]. 

### 2.6. Kinetic Modeling

An important role is played by the chemical architecture of any polymeric network in its swelling and drug release behavior. The dissolution media is penetrated through the micro-pathways into the polymeric network and thus dissolve the drug. After that, the dissolution media containing drug contents migrate to the surface of the polymeric network and the process is continued. The “r^2^” values of all formulations of the developed hydrogel are indicated in Table 3. Comparing the “r^2^” values, we can see that all formulations followed first order of kinetics, representing concentration-dependent release. Similarly, the “r^2^” values of the Higuchi model demonstrated diffusion-based release because the “r^2^” values were >0.5. In the case of Korsmeyer–Peppas, the “n” values for all formulations were obtained higher than 0.5, demonstrating non-Fickian behaviors [47].

### 2.7. FTIR Analysis

**Figure 4:**
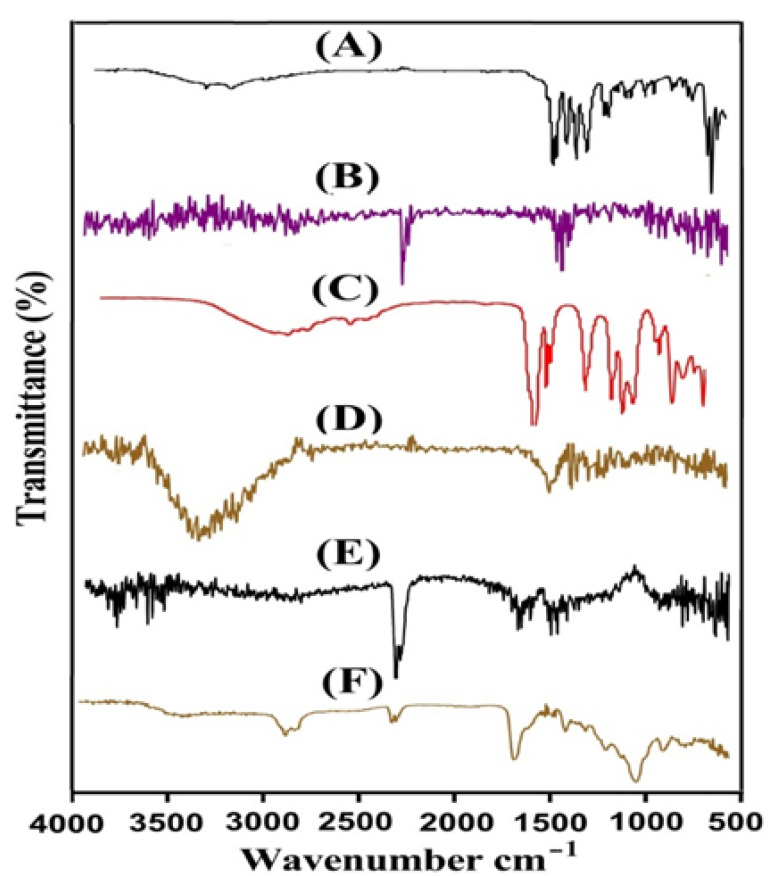
FTIR spectra of (**A**) Cs, (**B**) Sps, (**C**) Aca, (**D**) unloaded Cs/SpsCpAca hydrogel, (**E**) Ibu, and (**F**) loaded Cs/SpsCpAca hydrogel.

The structural arrangement of Cs, Sps, Aca, the unloaded hydrogel, Ibu, and the loaded hydrogel was investigated by FTIR analysis. Cs (Figure 4A) indicated the stretching vibration of −OH and N–H by its prominent peaks within the range of 3500–3000 cm^−1^, where overlapping of –OH and N–H occurred. The amide group revealed the stretching vibration by a band at 1627 cm^−1^. Two different peaks at 1389 and 1427 cm^−1^ were assigned to C–O and –OH overlapping, indicating the presence of a carboxyl group. The stretching vibration of the sulfate group (S=O) was determined by a peak at 1248 cm^−1^. Crispim et al. (2012) [48] and Ikrama et al. (2018) reported the same peaks of Cs in the same range as reported in the current study, which further supports our study [49]. Similarly, Sps revealed FTIR spectra (Figure 4B) by peaks at 1397 and 1510 cm^−1^, representing the symmetric and asymmetric vibration of the SO_3_ group. A peak at 642 cm^−1^ indicated the C-H stretching vibration [50]. Likewise, distinctive bands at 1298, 1610, and 3002 cm^−1^ depicted the –C=O, –C–C, and –CH_2_ stretching vibration, respectively, as revealed by FTIR spectra of Aca (Figure 4C) [51]. A fluctuation was seen in the positions of functional groups of polymers and monomer in the FTIR spectra of unloaded hydrogel (Figure 4D). The prominent peaks of Cs at 1627, 1427 cm^−1^, Sps at 1397, 1510 cm^−1^, while Aca at 1298, 1610 cm^−1^ were shifted to 1515, 1440, 1413, 1490, 1312, and 1580 cm^−1^, respectively, due to the electrostatic interaction among them. A few peaks such as 1389 cm^−1^ (Cs), 642 cm^−1^ (Sps), and 3002 cm^−1^ (Aca) disappeared, while some new peaks were formed. This shifting, disappearance, and formation of new bands indicated the synthesis of a new polymeric drug carrier system of hydrogel. Similarly, Ibu (Figure 4E) perceived the carbonyl stretching vibration of isopropanoic acid groups by a peak within the range of 1738–1832 cm^−1^, whereas the stretching vibration of CH, OH, and COOH was observed by peaks at 3492, 3258, and 2940 cm^−1^. Likewise, the C–C stretching vibration was shown by a peak at 1498 cm^−1^ [52]. A minor change was observed in certain peaks of Ibu in the FTIR spectra of drug-loaded hydrogel (Figure 4F). The distinct peaks of Ibu shifted from 1738 and 1498 cm^−1^ to 1710 and 1488 cm^−1^ in drug-loaded hydrogel, indicating the successful loading of Ibu by the fabricated hydrogel. Thus, no chemical interaction was observed between the Ibu and hydrogel contents [49].

### 2.8. SEM

**Figure 5:**
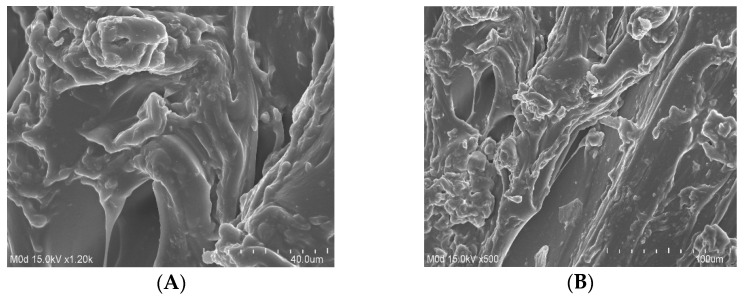

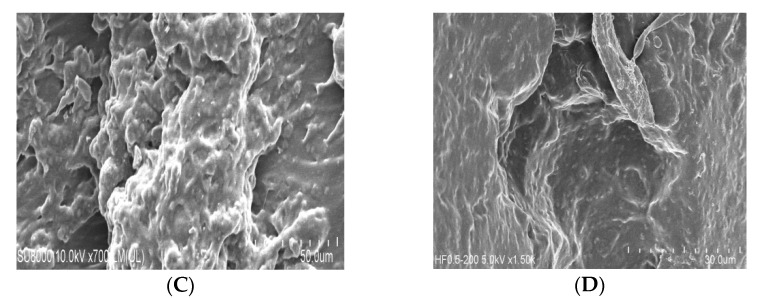
Scanning electron microscopy of different formulations of the Cs/SpsCpAca hydrogel with the increasing concentration of (**A**,**B**) Cs, (**C**) Sps, and (**D**) Aca.

The surface morphology of the formulated hydrogel was investigated by SEM. A hard and irregular surface with a few large pores was perceived in the prepared hydrogel, as indicated in Figure 5. The hard and rough surface of the developed hydrogel presented strong crosslinking among Cs, Sps, and Aca, whereas the large pores indicated water penetration into the hydrogel networks. Water penetrates through the pores into the hydrogel, due to which swelling and the loading of the drug occur. The greater the number of pores on the surface of the hydrogel, the greater the swelling of the hydrogel, thus increasing the drug loading, and vice versa [32].

### 2.9. TGA 

**Figure 6:**
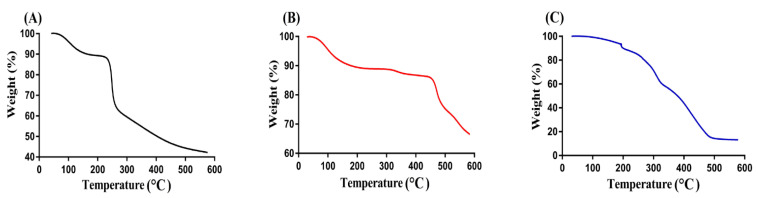
TGA thermogram of (**A**) Cs, (**B**) Sps, and (**C**) Cs/SpsCpAca hydrogel.

A TGA thermogram was conducted with the purpose of evaluating the thermal stability of Cs, Sps, and Cs/SpsCpAca hydrogel, as indicated in Figure 6. The weight reduction in polymers and developed hydrogels occurred at three different stages. At the 1st stage, a weight reduction of 17% was perceived by the TGA thermogram of Cs (Figure 6A) within a temperature range of 98–252 °C due to the moisture loss and anhydride formation by polymer chains. At the 2nd stage, a further weight reduction of 32% was detected as the temperature approached 352 °C, which may be attributed to the primary degradation of carboxylate and sulfonate groups of the polymer. At the 3rd stage, degradation of Cs started up to entire degradation with a further weight loss of 10% [53]. Similarly, during the 1st stage, the TGA thermogram of Sps (Figure 6B) presented a weight reduction of 8% at 250 °C. At the 2nd stage, a 5% decrease in weight was observed with a further enhancement in temperature up to 460 °C, while at the 3rd stage, degradation of Sps was started with a weight reduction of 20% [54]. At the 1st stage, the TGA thermogram of Cs/SpsCpAca hydrogel (Figure 6C) indicated a weight reduction of 35% within a temperature range of 98–310 °C. Likewise, at the 2nd stage, a further decrease of 40% in weight was seen at 490 °C, which is attributed to the decomposition of functional groups of both Cs and Sps. At the 3rd stage, a weight reduction of 3% was seen finally at 495 °C, and then degradation of the developed hydrogel was started. The TGA thermogram of Cs, Sps, and fabricated hydrogel indicated that the degradation half-life of Cs/SpsCpAca hydrogel (t1/2 = 495 °C) was greater than the degradation half-lives of Cs and Sps, i.e., Cs (t1/2 = 352 °C) and Sps (t1/2 = 460 °C), respectively. The increase in thermal stability of Cs and Sps was due to the crosslinking and electrostatic interaction of the hydrogel contents, which led to the synthesis of a stable network of hydrogel [37,55]. 

### 2.10. DSC Analysis

**Figure 7:**
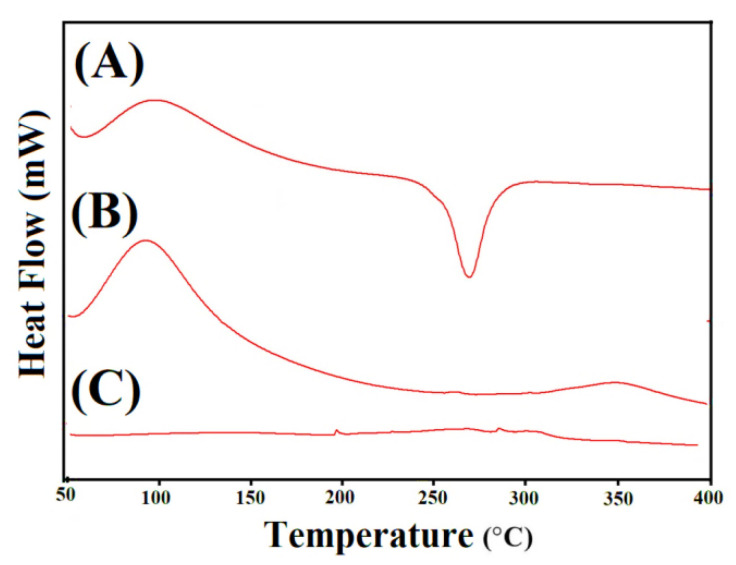
DSC thermogram of (**A**) Cs, (**B**) Sps, and (**C**) Cs/SpsCpAca hydrogel.

DSC analysis was conducted for Cs, Sps, and the formulated hydrogel to reveal the changes in thermal stability of Cs and Sps after the crosslinking and development of hydrogel. The DSC thermogram of Cs (Figure 7A) presented an endothermic peak within the 48–68 °C range of temperature, followed by dehydration and elimination of other volatile constituents. A strong endothermic peak was observed at 260 °C, which indicated degradation of the polymer chain, whereas two exothermic peaks were seen at 98 and 268 °C, respectively. The peak at 98 °C was assigned to glass transition temperature Tg, while the peak at 268 °C revealed oxidative degradation of the polymer [56]. Similarly, Sps presented two endothermic peaks at 59 and 262 °C. The endothermic peak at 59 °C was attributed to glass transition, whereas the other endothermic peak at 262 °C indicated Sps degradation (Figure 7B). Likewise, two exothermic peaks were depicted at 98 and 350 °C by the DSC thermogram of Sps. The DSC thermogram of the developed polymeric network of hydrogel (Figure 7C) exhibited two exothermic peaks at 198 and 284 °C. The first peak was the exothermic peak of Cs shifting from 268 °C to a 198 °C peak of prepared hydrogel, whereas the other peak assigned to Sps moved from 350 °C to 284 °C in developed hydrogels, indicating the high constancy and stability of the prepared hydrogels [57]. Similarly, an endothermic peak at 312 °C was depicted by DSC thermogram of the developed hydrogel and indicated the shifted endothermic peak of Cs from 260 °C to 312 °C, respectively. Hence, we can conclude that thermal stability of the developed hydrogel was greater than Cs and Sps [58,59]. 

### 2.11. PXRD Analysis

**Figure 8:**
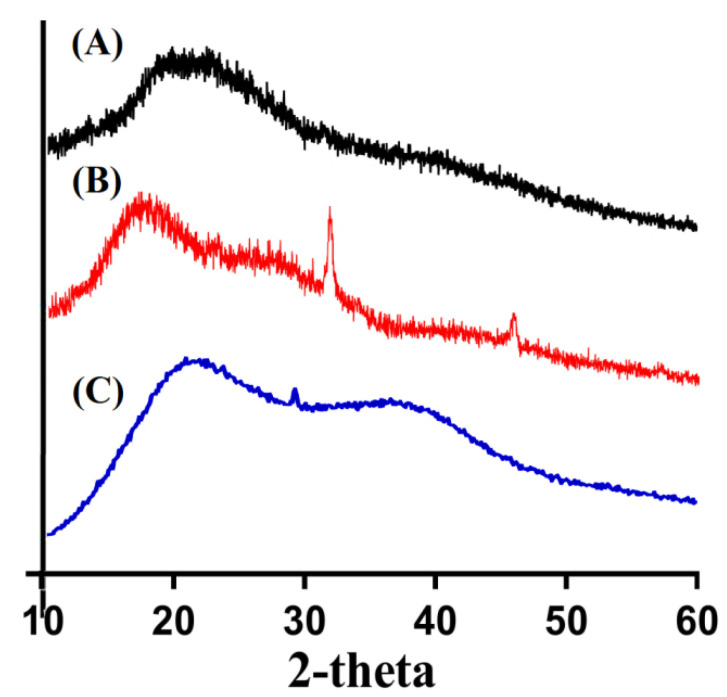
PXRD of (**A**) Cs, (**B**) Sps, and (**C**) Cs/SpsCpAca hydrogel.

PXRD was investigated for Cs, Sps, and Cs/SpsCpAca hydrogel in order to examine their crystallinity, as indicated in Figure 8. PXRD of Cs (Figure 8A) presented minor sharp and broad peaks at 2*θ* = 19.08°, 21.80°, and 27.41° [37], where PXRD of Sps (Figure 8B) revealed high intense crystalline peaks at 2*θ* = 32.70° and 47.28°, respectively [60]. The crystallinity of pure Cs and Sps was reduced/disappeared by fabricated networks of hydrogel, as shown in Figure 8C. Due to strong crosslinking among the polymers and monomer, sharp peaks of polymers disappeared and thus a stable hydrogel network was formed. Lee et al. (2018) prepared amphiphilic hydrogel based on poly(l-lactide) and chondroitin sulfate and demonstrated a reduction in the high crystalline and intense sharp peaks of copolymers by the developed hydrogel, which further supports our hypothesis [61]. 

## 3. Conclusions

The recent investigation was based on the development, characterization, and evaluation of Cs/SpsCpAca hydrogel for the controlled release of ibuprofen. Sol-gel analysis demonstrated very high gel and low sol fractions. High significant swelling and drug release were found at pH 7.4 as compared to pH 1.2 and 4.6, representing the pH-responsive nature of the fabricated hydrogel. FTIR confirmed the development and loading of the drug by the developed hydrogel. SEM indicated the hard and irregular surface of the prepared hydrogel. Similarly, TGA and DSC thermogram demonstrated the greater thermal stability of the polymeric hydrogel, while a decrease in crystallinity of Cs and Sps after polymerization was perceived by PXRD. Hence, we conclude that the newly prepared polymeric pH-sensitive hydrogel ensures excellent swelling, drug release, and high stability, and thus could be used as a potential and promising carrier for the controlled delivery of Ibu.

## 4. Materials and Methods

### 4.1. Materials

Cs was procured from Sigma-Aldrich (St. Louis, MO, USA). Sps was acquired from Alfa Aesar (Ward Hill, MA, USA). Ibu and Aca were purchased from Acros (Carlsbad, CA, USA), while ammonium persulfate (Aps) was acquired from Showa (Tokyo, Japan). N′,N′-Methylene bisacrylamide (Mba) was procured from Alfa Aesar (Lancashire, UK). 

### 4.2. Fabrication of Polymeric Hydrogels

The fabrication of chondroitin sulfate/sodium polystyrene sulfonate–co-poly(acrylic acid) (Cs/SpsCpAca) hydrogel was performed by the free radical polymerization technique. The composition of the developed hydrogel is indicated in Table 4. The crosslinking of Cs and Sps with Aca was carried out by Mba in the presence of Aps. Accurate weighed amount of Cs was taken and dissolved in deionized distilled water while continuously stirring at 50 °C with 50 rpm. Similarly, a specific quantity of Aps and Sps was taken and dissolved in a required quantity of deionized distilled water. Aps was added into the Sps solution, and then poured into the solution of Cs after proper mixing. Aca was already in liquid form, hence added dropwise into the polymers and initiator mixture. The mixture was continuously stirred. Mba was dissolved in a mixture of water and ethanol. Finally, solution of Mba was poured into the stirred mixture. A transparent solution was formed. Nitrogen gas was passed through the transparent solution in order to eliminate dissolved oxygen. After that, the transparent solution was transferred into glass molds, which were placed in the water bath at 55 °C for the initial 2 h, and then the temperature was enhanced up to 65 °C for the next 22 h. The prepared gel was cut into 8 mm size discs and washed by a mixture of ethanol and water in order to remove any impurity attached to the surface of the discs. The prepared gel discs were placed at room temperature for 24 h and then subjected to drying in vacuum oven at 40 °C for 7 days. The prepared discs were assessed for further experiments. 

### 4.3. Sol-Gel Analysis

The amount of reactants consumed in the fabrication of Cs/SpsCpAca hydrogels was evaluated by sol-gel analysis. Hence, accurate weighed discs of hydrogel were placed in a Soxhlet apparatus which already contained an appropriate amount of deionized distilled water. The extraction process was performed for 12 h. After that, the discs of hydrogel were taken out and placed at 40 °C in the vacuum oven for dehydration. The dried discs of hydrogel were measured again [62]. Sol-gel analysis was determined by the given equations:

(1) $$\mathrm{Sol}\;\mathrm{fraction}\;\%=\frac{{\;T}_{1}-{\;T}_{2}}{{\;T}_{2}}\times 100$$

(2) Gel fraction=100−Sol fraction

T_1_ indicates the initial weight of dried hydrogel disc before the extraction process, while T_2_ represents the final weight after the extraction. 

### 4.4. Porosity Study

Solvent replacement technique was employed for the estimation of porosity of the Cs/SpsCpAca hydrogel. Initially, weighed dried discs of hydrogel (D_1_) were taken and then immersed in the absolute ethanol for 72 h. After equilibrium swelling, discs were removed, blotted with filter paper to eliminate the attached ethanol from the surface of the hydrogel discs, and then weighed again (D_2_) on weighing balance [63]. Percent porosity of the fabricated hydrogel was determined by the given equation:

(3) $$(\%)\;\mathrm{Porosity}=\frac{D_{2}-D_{1}}{\rho V}\times 100$$

ρ is the density of absolute ethanol, whereas V is the swelling volume of hydrogel discs. 

### 4.5. Dynamic Swelling

The swelling dynamics of the fabricated hydrogel was investigated in three different pH values, i.e., pH 1.2, 4.6, and 7.4, which were used as swelling media. Hence, weighed discs of the developed hydrogels were soaked in the respective pH buffer solution at 37 °C. After a regular interval of time, hydrogel discs were taken out and blotted off cautiously to eliminate liquid attached to the surface of the hydrogel discs. Later, the swelled discs were weighed again on weighing balance and immersed back in the respective buffer solution. This action was continued until no further increase in weight of the swelled hydrogel discs was observed [64]. The swelling index was determined by the given equation:

(4) $$\left(q\right)=\frac{{\;L}_{2}}{L_{1}}$$

(q) represents the dynamic swelling, L_1_ indicates the initial weight of dried hydrogel disc before swelling, and L_2_ shows the final weight after swelling at time t. 

### 4.6. Polymer Volume Fraction

Polymer volume fraction is the fraction of polymer of the hydrogel in swelled state, which is represented by V2,s. Equilibrium volume swelling (Veq) data were employed for the estimation of polymer volume fraction at pH 1.2, 4.6, and 7.4, respectively [35]. Hence, the given equation was used for the estimation of polymer volume fraction of developed hydrogel in fully swollen state at respective pH values:

(5) $$V2,s=\frac{1}{\mathrm{Veq}}$$

### 4.7. Drug Loading

Loading of drug by the developed hydrogel was performed by diffusion and absorption method. Hence, 1% solution of the drug was formed in phosphate buffer solution of pH 7.4 at room temperature. Dried hydrogel discs were weighed initially and then immersed in the drug solution for 72 h. After equilibrium swelling and loading of drug, discs were removed from the drug solution. The swelled drug-loaded discs of hydrogel were washed by deionized distilled water to remove the entrapped drug attached to the surface of the hydrogel discs. Finally, the loaded discs were placed at 40 °C in the vacuum oven for dryness.

Estimation of loaded contents of drug by the prepared hydrogel was carried out by two methods. (I) Extraction method: In this method, dried loaded hydrogel discs were placed in 25 mL phosphate buffer solution of pH 7.4. After a specific period of time, samples were collected, and medium was replaced by fresh medium of the same buffer solution with the same concentration. This process was continued until entire drug was eliminated completely from the hydrogel discs. The samples were then analyzed on UV–Vis spectrophotometer (U-5100,3J2-0014, Tokyo, Japan) at λ_max_ 222 nm in a triplicate [54].

(II) Weight method: In this method, weight difference was determined between the loaded and unloaded discs of hydrogel. Hence, weight of unloaded discs of hydrogel was subtracted from the weight of loaded discs of hydrogel [32,65]. Weight difference was determined by the given equation in order to estimate the loaded content of the drug by the developed hydrogel:

Drug-loaded quantity = B_L_ − B_UL_ (6)

B_L_ indicates the weight of drug-loaded discs of hydrogel and B_UL_ shows the weight of unloaded discs of hydrogel.

### 4.8. Dissolution Study

Dissolution study was carried out for prepared hydrogels at three different pH values, i.e., pH 1.2, 4.6, and 7.4. This experiment was performed by immersing the weighed loaded hydrogel discs in 900 mL buffer solution of the respective pH value while using USP dissolution apparatus type II (USP dissolution (Sr8plus Dissolution Test Station, Hanson Research, Chatsworth, CA, USA)) at 37 ± 0.5 °C and 50 rpm. A sample of 5 mL was taken after a specific interval of time and fresh buffer solution of the same concentration was added back to maintain the sink condition constant. The samples were analyzed on UV–Vis spectrophotometer (U-5100,3J2-0014, Tokyo, Japan) in a triplicate at λ_max_ 222 nm [66].

### 4.9. Kinetic modeling

Various kinetic models such as zero order, first order, Higuchi, and Korsmeyer–Peppas were computed for release data of various formulations of hydrogels in order to evaluate the release mechanism of the drug from the prepared hydrogels [67].

### 4.10. FTIR Analysis

The spectral analysis of Cs, Sps, Aca, Ibu, the unloaded, and the loaded formulated hydrogel was performed by Attenuated Total Reflectance FTIR (NICOLET 380 FTIR (Thermo Fisher Scientific, Ishioka, Japan)) FTIR. The samples were crushed and then FTIR spectrum was performed within the range of 4000–500 cm^−1^ [68].

### 4.11. SEM

The surface morphology of the developed hydrogel was performed by SEM (JEOL, Tokyo, Japan). Gold was used for sputtering the samples, which were then placed on aluminum stub. Scanning of samples was carried out by various magnifications [69].

### 4.12. Thermal Properties

Thermal properties of polymers and prepared hydrogel were evaluated by TGA (PerkinElmer Simultaneous Thermal Analyzer STA 8000, PerkinElmer Ltd., Buckinghamshire, UK) and DSC (PerkinElmer DSC 4000, Waltham, MA, USA). TGA was carried out for Cs, Sps, and developed hydrogel to determine their thermal stability with the enhancement in temperature up to optimum level. Hence, samples of 5 mg weight were placed in a platinum pan, which was attached to a microbalance. TGA thermogram for all samples was carried out at a heat rate of 20 °C/min. Temperature was fixed within the range of 40–600 °C, respectively. Nitrogen flow rate was kept 20 mL/min throughout the experiment [70]. Similarly, DSC analysis was performed for polymers and formulated hydrogel with the purpose to check their thermal stability. Hence, samples of 5 mg were taken and heated within temperature range of 50 to 400 °C at a rate of 20 °C/min. The rate of nitrogen flow was kept 20 mL/min throughout the experiment [71].

### 4.13. PXRD Analysis

The nature of the Cs, Sps, and prepared hydrogel was investigated by PXRD (XRD-6000 Shimadzu, Tokyo, Japan). PXRD analysis of samples was performed within angle diffraction of 10 to 60° with 2θ 2°/min [72].

### 4.14. Statistical Analysis

SPSS Statistic software 22.0 (IBM Corp, Armonk, NY, USA) was used for the statistical analysis. The variations between the tests were estimated by using Student’s t-Test. The obtained results were found significant statistically, because *p*-value was found less than 0.05 (*p* < 0.05).

## Figures and Tables

**Table 1:** Dynamic swelling and polymer volume fraction of Cs/SpsCpAca hydrogels.

Formulation Code	Dynamic Swelling up to 72 h			Polymer Volume Fraction		
	pH 1.2	pH 4.6	pH 7.4	pH 1.2	pH 4.6	pH 7.4
N-1	3.58 ± 0.20	5.42 ± 0.18	10.78 ± 0.26	0.279	0.184	0.092
N-2	3.72 ± 0.18	6.20 ± 0.22	11.80 ± 0.19	0.268	0.161	0.084
N-3	3.80 ± 0.23	6.60 ± 0.20	12.30 ± 0.17	0.263	0.151	0.081
N-4	3.58 ± 0.20	5.42 ± 0.18	10.78 ± 0.26	0.279	0.184	0.092
N-5	3.76 ± 0.16	6.33 ± 0.23	11.94 ± 0.14	0.265	0.157	0.083
N-6	3.84 ± 0.22	6.72 ± 0.21	12.45 ± 0.22	0.260	0.148	0.080
N-7	3.45 ± 0.25	5.13 ± 0.28	08.94 ± 0.27	0.289	0.194	0.111
N-8	3.58 ± 0.20	5.42 ± 0.18	10.78 ± 0.26	0.279	0.184	0.092
N-9	3.88 ± 0.19	6.78 ± 0.25	12.90 ± 0.24	0.257	0.147	0.077

**Table 2:** Drug loading of Cs/SpsCpAca hydrogels.

Formulation Code	Drug Loaded (mg)/500 mg of Dry Gels	
	Weight Method	Extraction Method
N-1	130.8 ± 0.9	128.9 ± 1.1
N-2	171.6 ± 0.8	170.1 ± 0.9
N-3	198.1 ± 0.9	196.8 ± 1
N-4	130.8 ± 0.9	128.9 ± 1.1
N-5	180.2 ± 1	179.1 ± 0.9
N-6	205.2 ± 1	203.2 ± 0.9
N-7	92.7 ± 1	91.5 ± 1
N-8	130.8 ± 0.9	128.9 ± 1.1
N-9	212.4 ± 0.9	210.9 ± 0.9

**Table 3:** Kinetic modeling release of Ibu from Cs/SpsCpAca hydrogels.

F. Code	Zero Order r^2^	First Order r^2^	Higuchi r^2^	Korsmeyer–Peppas	
				r^2^	n
N-1	0.9477	0.9864	0.8563	0.9740	0.7738
N-2	0.9554	0.9930	0.9910	0.9846	0.7093
N-3	0.9710	0.9980	0.9947	0.9918	0.5937
N-4	0.9477	0.9864	0.8563	0.9740	0.7738
N-5	0.8829	0.9953	0.9729	0.9687	0.5687
N-6	0.8946	0.9827	0.9795	0.9817	0.5250
N-7	0.9528	0.9903	0.9840	0.9677	0.5558
N-8	0.9477	0.9864	0.8563	0.9740	0.7738
N-9	0.9566	0.9948	0.9915	0.9877	0.7498

**Table 4:** Feed ratio scheme for formulation of Cs/SpsCpAca hydrogels.

F. Code	Polymer Cs g/100 g	Polymer Sps g/100 g	Monomer Aca g/100 g	Initiator Aps g/100 g	Crosslinker Mba g/100 g
N-1	0.24	0.24	20	0.4	0.4
N-2	0.48	0.24	20	0.4	0.4
N-3	0.72	0.24	20	0.4	0.4
N-4	0.24	0.24	20	0.4	0.4
N-5	0.24	0.52	20	0.4	0.4
N-6	0.24	0.80	20	0.4	0.4
N-7	0.24	0.24	16	0.4	0.4
N-8	0.24	0.24	20	0.4	0.4
N-9	0.24	0.24	24	0.4	0.4

## Data Availability

Not applicable.
